# Application of the thermostable β-galactosidase*, BgaB,* from *Geobacillus stearothermophilus* as a versatile reporter under anaerobic and aerobic conditions

**DOI:** 10.1186/s13568-017-0469-z

**Published:** 2017-09-06

**Authors:** Torbjørn Ølshøj Jensen, Ivan Pogrebnyakov, Kristoffer Bach Falkenberg, Stephanie Redl, Alex Toftgaard Nielsen

**Affiliations:** The Novo Nordisk Foundation Center for Biosustainability, Technical University of Denmark, Kemitorvet Building 220, 2800 Kongens Lyngby, Denmark

**Keywords:** Genetic reporter, Anaerobic genetics, Thermophile, Thermostable enzyme

## Abstract

Use of thermophilic organisms has a range of advantages, but the significant lack of engineering tools limits their applications. Here we show that β-galactosidase from *Geobacillus stearothermophilus* (BgaB) can be applicable in a range of conditions, including different temperatures and oxygen concentrations. This protein functions both as a marker, promoting colony color development in the presence of a lactose analogue S-gal, and as a reporter enabling quantitative measurement by a simple colorimetric assay. Optimal performance was observed at 70 °C and pH 6.4. The gene was introduced into *G. thermoglucosidans*. The combination of BgaB expressed from promoters of varying strength with S-gal produced distinct black colonies in aerobic and anaerobic conditions at temperatures ranging from 37 to 60 °C. It showed an important advantage over the conventional β-galactosidase (LacZ) and substrate X-gal, which were inactive at high temperature and under anaerobic conditions. To demonstrate the versatility of the reporter, a promoter library was constructed by randomizing sequences around −35 and −10 regions in a wild type *groES* promoter from *Geobacillus* sp. GHH01. The library contained 28 promoter variants and encompassed fivefold variation. The experimental pipeline allowed construction and measurement of expression levels of the library in just 4 days. This β-galactosidase provides a promising tool for engineering of aerobic, anaerobic, and thermophilic production organisms such as *Geobacillus* species.

**Electronic supplementary material:**

The online version of this article (doi:10.1186/s13568-017-0469-z) contains supplementary material, which is available to authorized users.

## Introduction

Economically feasible production of biofuels and biochemicals using microbial cell factories is becoming an increasingly important challenge in the transition towards a sustainable society. Development and optimization of suitable production microorganisms is essential to meet this challenge. Direct engineering of the metabolic pathways of these microorganisms is a recognized method for improving properties and performances. Tuning gene expression to perform metabolic optimization rather than substantial overexpression or inactivation of genes is thus far more appreciated.

Today, strain development for production of many biochemicals is based on metabolic engineering of few mesophilic organisms, such as *Escherichia coli* and *Saccharomyces cerevisiae* (Fisher et al. 2014; Otero et al. 2013; Woolston et al. 2013). The choice of production host has largely been determined by the availability of efficient engineering tools that enable rapid strain development. Besides being convenient, these organisms are not necessarily the best-suited hosts for robust and low cost production of chemicals.

Thermophilic fermentation has several advantages compared to conventional mesophilic fermentation, including: (i) metabolic rates are typically higher at elevated temperatures; (ii) thermophiles are robust and contain thermotolerant enzymes; (iii) thermophilic organisms generally have a low cellular growth yield, hence more substrate carbon is directed towards the product; (iv) thermophilic fermentations are less prone to contaminations by mesophiles; (v) growth at high temperature facilitates recovery of volatile products, for example ethanol (Payton 1984); (vi) fermentation at elevated temperatures reduces the requirement for cooling (Kuhad and Singh 1993; Payton 1984; Wiegel and Ljungdahl 1986). Examples of metabolic engineering of thermophiles have long been limited, but have recently started to emerge (Bhandiwad et al. 2014; Cripps et al. 2009; Shaw et al. 2008; van der Veen et al. 2013). However, genetic tools are often strain-specific (Klapatch et al. 1996; Mai et al. 1997; Mai and Wiegel 2000), and there is a need for a generalized toolbox that allows genetic manipulation and screening of thermophilic production strains.

In particular, the variety of genetic markers and reporters suited for thermophiles is very limited, although a few thermostable variants of green fluorescent protein (GFP) have recently been developed (Aliye et al. 2015; Kiss et al. 2009). A major limitation for applying the GFP variants is that the formation of the chromophores strictly requires oxygen, which restricts the use to aerobic systems (Piatkevich and Verkhusha 2011).

The enzyme β-galactosidase (EC 3.2.1.23), commonly named lactase, catalyzes the hydrolysis of the terminal non-reducing β-d-galactose residues in β-d-galactosides. For example, it catalyzes the hydrolysis of lactose into glucose and galactose. Several β-galactosidases have been isolated and characterized for the production of lactose-free milk products (Panesar et al. 2006). Thermostable β-galactosidases possess a considerable industrial potential due to their high activity at elevated temperatures (Chen et al. 2008; Pessela et al. 2003; Zeikus et al. 1998). Different thermostable β-galactosidases from bacteria, archaea, and fungi have been identified and characterized with industrial perspective, from species including: *Thermus* sp. (Ohtsu et al. 1998; Ulrich et al. 1972), *Geobacillus stearothermophilus* (Chen et al. 2008), *Thermotoga maritima* (Kim et al. 2004), *Thermoanaerobacter* sp. (Lind et al. 1989), *Bacillus coagulans* (Batra et al. 2002), *Pyrococcus woesei* (Daabrowski et al. 2000), *Rhizomucor* sp. (Shaikh et al. 1999), and *Talaromyces thermophilus* (Nakkharat and Haltrich 2006). The most studied β-galactosidase is derived from *E. coli*, notably a mesophile, and is encoded by *lacZ*. It has been used to elucidate the catalytic mechanism of this enzyme (Mahoney 1997), and it is frequently used in life sciences, since the active enzyme is easily detected when the lactose analogue 5-bromo-4-chloro-3-indolyl-β-d-galactopyranoside (X-gal) is cleaved forming an intense blue product. The color development requires the presence of oxygen, which limits its use to aerobic bacteria. The more recently developed thermostable dye, 3,4-cyclohexenoesculetin β-d-galactopyranoside (S-gal), does not require oxygen for development of a black product (Heuermann and Cosgrove 2001). Application of this dye would enable color-based selection in thermophilic microorganisms, both anaerobic and aerobic.

In this paper, we characterize the β-galactosidase encoded by *bgaB* from *G. stearothermophilus* for molecular applications in thermophiles and under anaerobic conditions. The *bgaB* gene has previously been applied as a reporter to monitor heat/stress response in *Bacillus subtilis* (Schrogel and Allmansberger 1997; Yuan and Wong 1995), as well as to gain expression profiles of various promoters in *Geobacillus kaustophilus* HTA426 (Suzuki et al. 2013), thus it is considered suited for the purpose. Since *G. thermoglucosidans* is a facultative anaerobe and capable of growing at a broad range of temperatures, it is a suitable platform for testing this and other systems, which require various conditions. We demonstrate *bgaB* as an efficient tool for colony screening of thermophilic aerobic and anaerobic microorganisms. Furthermore by showing its capacity in quantifying promoter strength in a randomized library we highlight the versatility of the tool.

## Materials and methods

### Strains, plasmids, and primers

The strains and plasmids used in this study are listed in Table 1. The primers used for the constructs are listed in Table 2.

**Table 1:** List of strains and plasmids

Name	Relevant characteristics	Reference
Strains		
*E. coli* TOP10	F− *mcrA* Δ(*mrr*-*hsd*RMS-*mcr*BC) Φ80*lac*ZΔM15 Δ *lac*X74 *rec*A1 *ara*D139 Δ (*ara leu*) 7697 *gal*U *gal*K *rps*L (StrR) *end*A1 *nup*G	Thermo Fisher Scientific, USA
*E. coli* TOP10: pUC19-*bgaB*	*E. coli* harboring the plasmid pUC19-*bgaB*	This study
*G. stearothermophilus* DSM2027	Source of *bgaB* gene	DSMZ GmbH, Germany
*G. thermoglucosidans* C56-YS93	Wild type isolate	*Bacillus* Genetic Stock Center, Ohio State University, USA
*G. thermoglucosidans:* pUCG18P5*bgaB*	Strain harboring pUCG18 with P5::*bgaB*	This study
Plasmids		
pUC19	General *E. coli* cloning vector; contains ampicillin resistance gene	Yanisch-perron et al. (1985)
pUC19*bgaB*	pUC19Δl*acZα* plasmid expressing the *bgaB* gene under control of the pLac promoter.	This study
pUCG18	*E. coli*/*Geobacillus* shuttle vector; AmpR, KanR. Used to construct a library of promoters in *E. coli*	Taylor et al. (2008)
pUCG18P5*bgaB*	Template for promoter library	This study
pMTL61110	*E. coli*/*Geobacillus* shuttle vector; AmpR, KanR. Used for expression in *G. thermoglucosidans*	Sheng et al. (2017)
pMTLP13B	pMTL61110 with P_13_-*bgaB*	This study
pMTLP24B	pMTL61110 with P_24_-*bgaB*	This study
pMTLP27B	pMTL61110 with P_27_-*bgaB*	This study
pMTLP13Z	pMTL61110 with P_13_-*lacZ*	This study
pMTLP24Z	pMTL61110 with P_24_-*lacZ*	This study
pMTLP27Z	pMTL61110 with P_27_-*lacZ*	This study

**Table 2:** List of primers used in this study

Name	Sequence 5′–3′	Target
Primers		
Beta-gal_fwd_USER	AGCTAUGAACGTTTTATCCTCAATTTGTTACGG	*bgaB*, with flanks for insertion into pUC19
Beta-gal_rev_USER	ACTACTCUAAACCTTCCCGGCTTCATC	
pUC19_fwd_USER	ATAGCUGTTTCCTGTGTGAAATTGTTATCCG	pUC19
pUC19_fwd_USER	AGAGTAGUTAAGCCAGCCCCGAC	
PNJ24b	AATTCGUAATCATGGTCATAGCTGTTTCC	pUCG18 backbone with terminator
PNJ27c	AGGGCTTUTGAGCCTTTCATTGAGGCTGTC	
PNJ23	ACCCGGGGAUCCTCTAG	pMTL backbone
PNJ24d	AATTCGUAATCATGGTCATATGGATACAGCG	
PNJ27b	AGGCTTUTGAGCCTTTCATTGAGG	Terminator of *groEL* gene forward
PNJ567	AGGAGGUCGTTTCCCATGAACGTTTTATCCTCAATTTGTTACGG	*bgaB*, with flanks for insertion into pUCG18
PNJ311	AAAGCCCUAAACCTTCCCGGCTTCATCATGCTCTC	
PNJ267	ACGAATUCGGCAAAACAACCGGCTCCTTTTGCTC	P_groES_ with CIRCE deleted
PNJ268	ACGATAGUTTTCGCCGTTCTTACACACTTATAATATTAATGAACTTCTTTCCGTTTTGC	
PNJ269	ACTATCGUTAAGGAGGTCGTTTCCCATGAGTAAAGGCGAAGAGCTGTTCAC	
PNJ388	ACACACUWWWWATATTAWWN_15_TTGCAANWWNNWWWTGCAAAAAAATAACTGTTTTTCTCTCCTAAAGAAGAAAG	P_groES_ with randomized sequences
PNJ389	AGTGTGUAAGAACGGCGAAAACTATCGTTAAG	
PNJ383	AGAGGCUACTCTCAAAAGGTCGGTTTAGACG	Terminator of *groEL* gene reverse
PNJ566	ACCTCCUTAACGATAGTTTTCGCC	P_groES_ reverse
PNJ672	AGGAGGUCGTTTCCCATGACCATGATTACGGATTCACTGG	*lacZ*
PNJ673	AAAGCCUTATTTTTGACACCAGACCAACTGG	

### Media and culture conditions

*Escherichia coli* was grown aerobically at 37 **°**C in lysogenic broth (LB), when necessary, supplemented with 100 µg/ml ampicillin and 250 µg/ml S-gal with 250 µg/ml ammonium ferric citrate. The mTGP medium [modified from Taylor et al. (2008)] was used to grow *G. thermoglucosidans* at 60 °C. It contained per liter: 17 g tryptone, 3 g soy peptone, 5 g NaCl, 2.5 g K_2_HPO_4_. After autoclavation, sterile solutions were added to final concentrations: 4 ml/l glycerol, 4 g/l sodium pyruvate, 0.59 mM MgSO_4_, 0.91 mM CaCl_2_, and 0.04 mM FeSO_4_; agar to 1.5% (w/v) was added to solidify the medium when needed. 12.5 µg/ml of kanamycin was used for selection of transformants.

For protein (BgaB) expression (under control of the *lacZ* promoter), a fresh *E. coli* culture was grown in LB media with appropriate antibiotics at 37 **°**C and 200 RPM of shaking until OD600 reached 0.5, then the expression was induced with IPTG (1 mM). The culture was allowed to further incubate for 3 h before determining the β-galactosidase activity.

### DNA manipulations

Genomic DNA was extracted using the Wizard^®^ Genomic DNA Purification Kit (Promega) according to producer’s specifications. Plasmid extractions were performed using NucleoSpin^®^ Plasmid EasyPure kit (Macherey–Nagel).

### Construction of the plasmids

Primers used in this study are listed in Table 2. All fragments were amplified with oligomers having uracil incorporated, using the Phusion U polymerase (Thermo Scientific). The plasmids and promoter library were constructed by the uracil-specific excision reagent (USER) cloning method (Geu-Flores et al. 2007; Nour-Eldin et al. 2006). In brief, 1 μl of 5× HF buffer (Thermo Scientific) and 1 U of USER™ enzyme mix (New England Biolabs, 1 U/ml) were added to 10 µl of the mixture of purified PCR products, plasmid backbone, or genes.

The reaction mixture was incubated for 25 min at 37 °C, followed by 25 min of incubation at a temperature optimized for annealing of the fragments for 25 min. 8 µl of water was added to the reactions, reaching a final volume of 20 µl. 5 µl diluted USER mixture was used to transform chemically competent *E. coli* TOP10 cells (Thermo Scientific) (Sambrook and Russell 2001).

### β-Galactosidase assay

The β-galactosidase activity was determined as described in Zhang and Bremer (1995), with the following modifications: hexadecyltrimethylammonium bromide and sodium deoxycholate were excluded from the substrate solution and the concentration of dibasic sodium phosphate in the permeabilization solution was 100 mM. The activity at different temperatures was determined by incubating the samples for 35 min at different temperatures, and stopping them by adding stop solution, then letting the samples cool before the absorbance (420 nm) was measured. The pH profiles (3.0–8.0) were determined at 70 °C in two buffer systems: citrate–phosphate buffer (200 mM) from pH 3.0 to 6.4 and sodium–phosphate buffer (200 mM) from pH 6.4 to 8.0. After adding the stop solution, the samples were centrifuged at 17,000*g* for 15 min, and absorbance of the supernatants was measured at 420 nm using a BioTek Synergy Mx Microplate Reader. The enzymatic activity was calculated following the equation below:

1 $$a = \frac{{Abs_{s} }}{{Abs_{c} \cdot V_{c} \cdot t}}$$

where *a* designates the enzyme activity (in Miller units), *Abs*_*s*_ is the absorbance at 420 nm of the sampled supernatant, *Abs*_*c*_ is the optical density at 600 nm of the sampled culture, *V*_*c*_ is the volume of the culture sampled and *t* is the reaction time. All samples were made in triplicates, unless stated otherwise.

For assays performed in deep 96-well microtiter plates (promoter library) the centrifugation was reduced to 4000*g* for 60 min. To compensate for possible presence of cell debris etc., absorbance at 550 nm was included and compensated in the activity calculations, which when multiplied by 1.75 estimates the light-scatter at 420 nm (Stephenson 2016). The extended version of Eq. (1) was used for the promoter library study.

2 $$a = \frac{{Abs_{420} - 1.75 \cdot Abs_{550} }}{{Abs_{c} \cdot V_{c} \cdot t}}$$

### Promoter library construction

The promoter of the *groES* gene (P_groES_), coding for a subunit of a chaperone complex, was placed upstream the *bgaB* gene (RefSeq WP_020755758.1) from *G. stearothermophilus* on the plasmid pUCG18 (Taylor et al. 2008). This was done using oligomers PNJ267, PNJ268, and PNJ269 in two steps to delete the CIRCE sequence responsible for the negative regulation of *groES* expression. After the β-galactosidase activity was observed on solid medium supplemented with S-gal, the library was constructed by whole plasmid-amplification using long DNA oligomers with partially randomized sequences (PNJ388 and PNJ389). To remove the parental plasmid, the amplicons were digested with *Dpn*I and isolated by gel-purification. The linearized plasmids were circularized by the USER-cloning method as described above. Transformants were selected on plates with ampicillin and S-gal. The black colonies were picked and cultivated in LB with ampicillin in deep 96 well plates at 37 °C overnight and used for inoculation the next day and subsequent activity measurements.

For the expression in *G. thermoglucosidans*, genes *bgaB* and *lacZ* were cloned into vector pMTL61110 (Sheng et al. 2017). The *bgaB* gene under P_groES_-derived promoters was amplified using primers PNJ267 and PNJ383 and cloned into the pMTL backbone, which was produced by PCR with primers PNJ23 and PNJ24d. The resulting plasmids were used as templates for PCR to derive backbones with respective promoters (primers PNJ27b and PNJ566), where the *lacZ* gene was cloned (amplified with primers PNJ672 and PNJ673 from genomic DNA of *E. coli* MG1655).

## Results

The thermostable β-galactosidase from *G. stearothermophilus* was initially cloned and expressed under control of the P_lac_ promoter in *E. coli*.

**Fig. 1:**
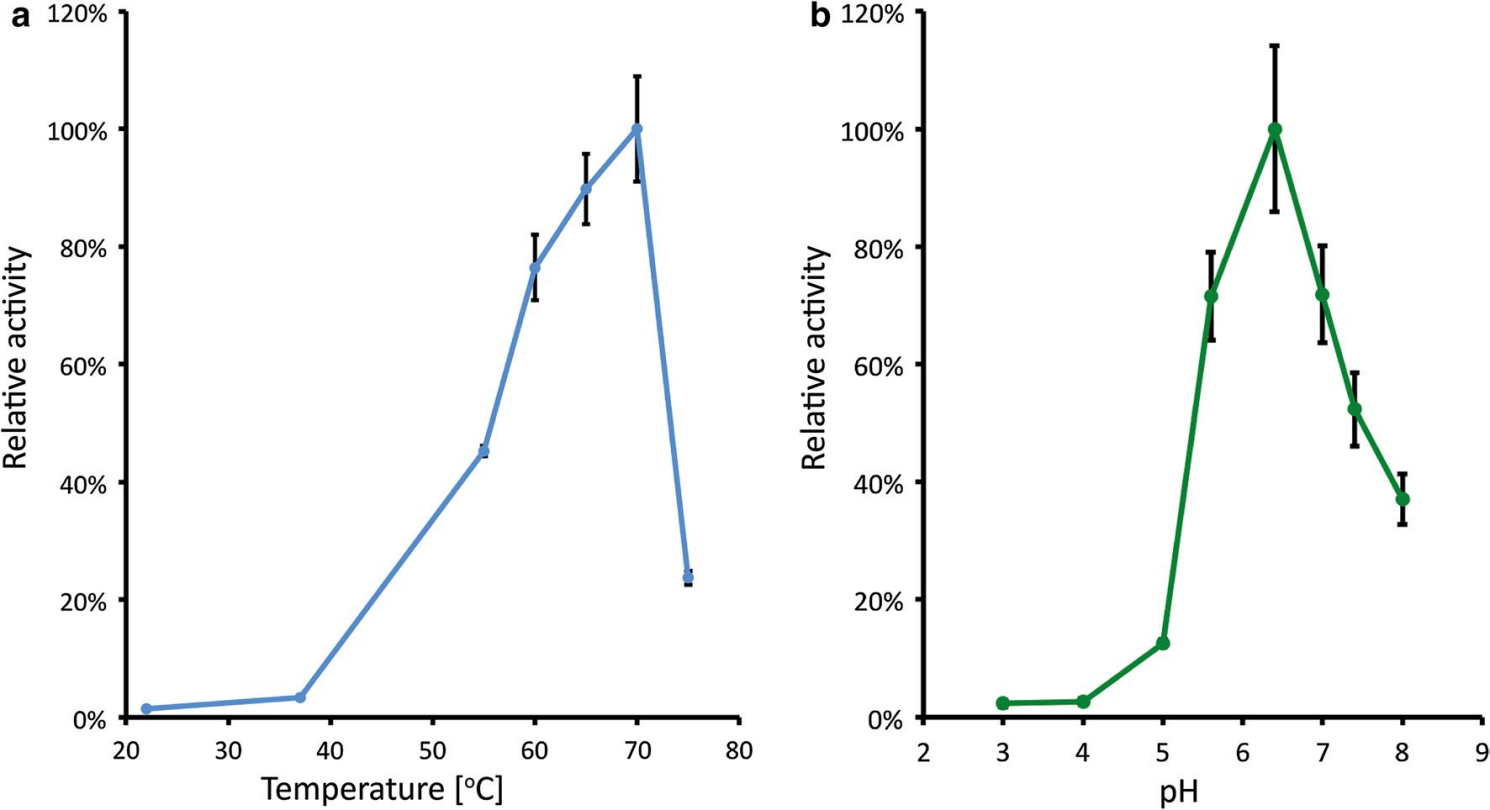
Temperature (**a**) and pH (**b**) profiles of the BgaB expressed in *E. coli*. The Y-axis designates the relative β-galactosidase activity at different conditions temperature (**a**) and pH (**b**). *Error bars* indicate standard deviation calculated based on triplicate experiments. pH profile was assessed at 70 °C

To evaluate the potential of this enzyme as a molecular tool under different conditions, its activity was measured in cell lysate at temperatures ranging from 22 to 75 °C and pH ranging from 3 to 8 (Fig. 1). At moderate temperatures the activity was low, while above 55 °C the activity was more pronounced. The highest activity (549.5 Miller units) was achieved at 70 °C. Increasing the temperature even further reduced the activity abruptly. The activity profile at various pH showed an optimum at pH 6.4. At higher pH, activity was still considerable, however, below pH 4 the activity was marginal.

### Quantification of gene expression by evaluating promoter library in *E. coli*

**Fig. 2:**
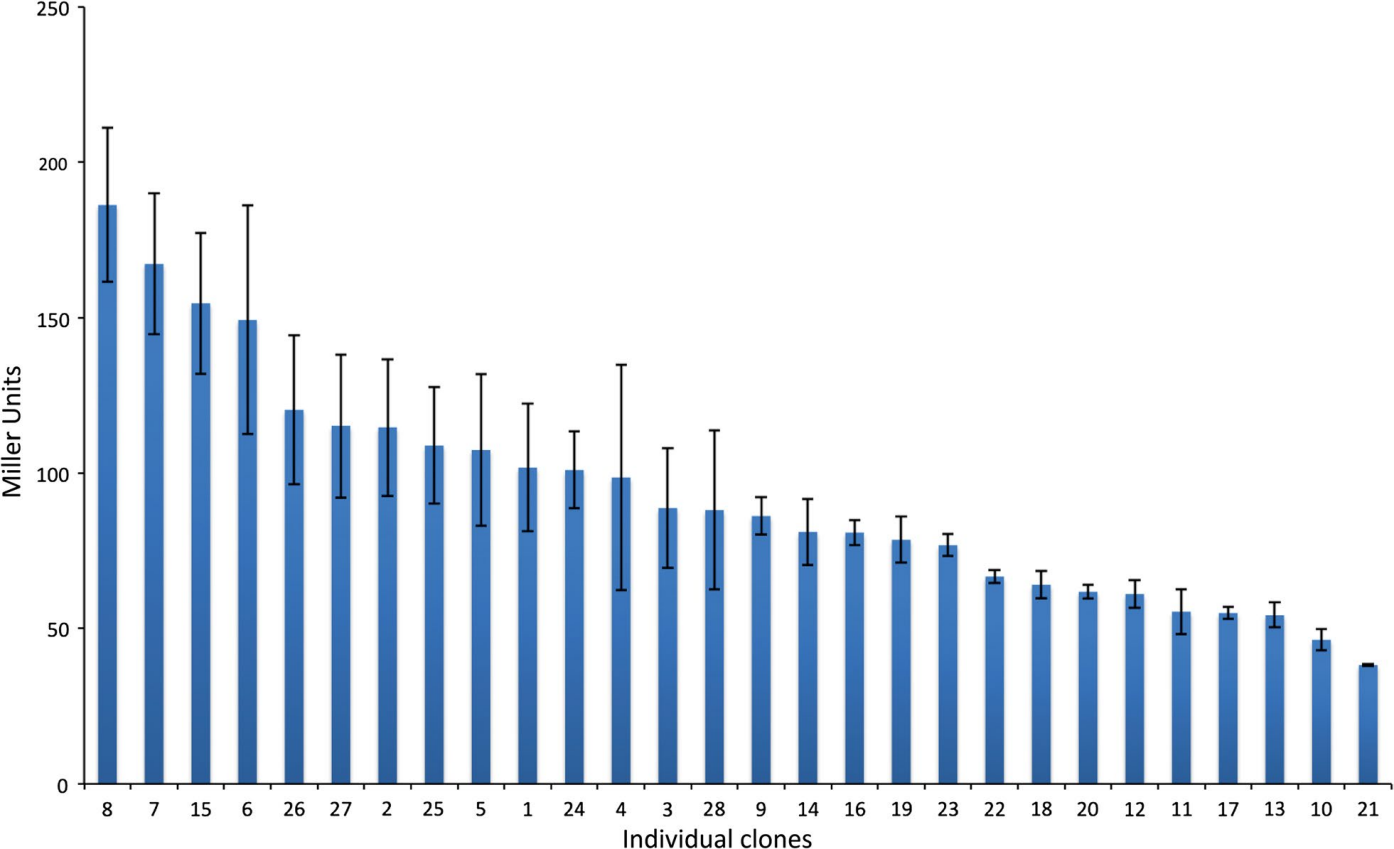
Promoter activities as measured by the expression levels of thermostable β-galactosidase in *E. coli*. The activity measurement was based upon 35 min of incubation at 60 °C. *Error bars* indicate standard deviation calculated from triplicate experiments

To demonstrate the applicability of *bgaB* as a reporter for quantitative measurements of expression levels, a promoter library was constructed using a method described by Jensen and Hammer (1998a, b). It includes the randomization of the *groES* promoter regions between −35 and −10 elements, while leaving these elements intact, as a way to vary promoter strength. In total, we obtained 28 clones with different promoter variants in *E. coli*. They were grown in 96-well microtiter plates to OD 2.5–4.0 and β-galactosidase activity was measured by incubating for 35 min at 60 °C (Fig. 2). The commonly used LacZ was not included as reference due to its marginal activity at the tested temperatures (Welsch et al. 2012). All variants displayed β-galactosidase activity. The highest activity (clone 8) measured was 186 Miller units (MU), whereas 38 MU was the lowest measured value (clone 21). This corresponds approximately to a fivefold difference. Of the 28 promoters, 19 showed an activity above 75 MU, whereas activity in 9 of the mutants was below 75 MU. Thus, the employed strategy proved successful for creating and selecting a wide range of expression variance.

As expected from the primer design, the alignment of promoter sequences shows great variation in the sequences upstream (8 bp), downstream (4 bp), and between (17 bp) the −35 and −10 regions (Additional file 1: Figure S1). There was a slight variation in GC content of the varied positions: the six mutants with highest activities had an average GC content of 31%, whereas the six mutants with lowest activity had an average of 26%. Promoter variant 12 stands out, since it had a point insertion downstream of the −10 sequence.

### Activity of thermostable β-galactosidase at different conditions

In the presence of Fe^3+^, β-galactosidase cleaves S-gal with a formation of a black product. Although β-galactosidase activity at 37 °C reached only 3% of the maximum, distinct black colonies are readily observed when it is expressed in *E. coli* (Additional file 1: Figure S2b). Color development was not observed in colonies of the negative control (Additional file 1: Figure S2a).

**Fig. 3:**
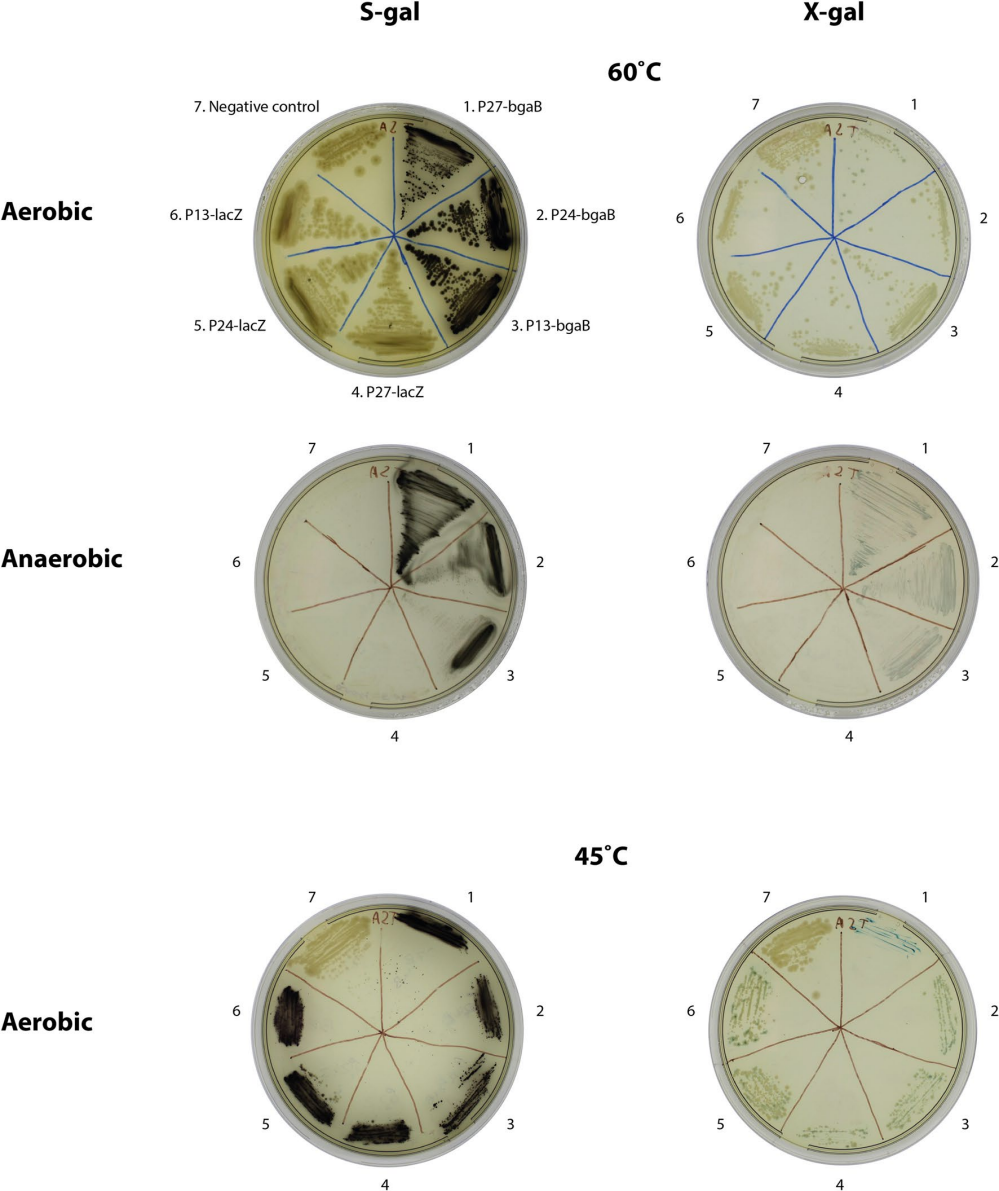
Expression of *bgaB* and *lacZ* in *G. thermoglucosidans* at 60 and 45 °C under aerobic and anaerobic conditions. The plates were supplemented with either S-gal or X-gal. Promoters P13, P24, and P27 of different strength were taken from the library (see Fig. 2)

We tested the applicability of BgaB and S-gal at different temperatures and oxygen levels. To this end, we expressed *bgaB* in *G. thermoglucosidans* under control of three promoters of different strengths (P_13_, P_24_ and P_27_) from the library described above (Fig. 3). The thermostable BgaB was compared to the commonly used LacZ β-galactosidase from *E. coli*, which was expressed under control of the same three promoters. The combination of BgaB with S-gal produced coloration in all circumstances, including 60 °C and under anaerobic conditions. On the contrary, LacZ was virtually inactive, and X-gal gave a much weaker color at high temperature. Since the color development from X-gal is known to require oxygen, we hypothesize that the observed blue color may be due to other compounds in the media or contamination with low levels of oxygen.

## Discussion

Application of *bgaB* as a versatile genetic reporter has been proven in mesophilic and thermophilic facultative aerobe bacteria and in mouse embryos in the presence of oxygen (Kishigami et al. 2006; Schrogel and Allmansberger 1997; Suzuki et al. 2013; Yuan and Wong 1995). Focusing on the applications of the *bgaB* gene under thermophilic and anaerobic conditions, we initially expressed it in *E. coli*. Optimal conditions for its activity were determined to be 70 °C and pH 6.4. The BgaB protein has previously been characterized by Chen et al. (2008) and Dong et al. (2011), aiming at applications in the dairy industry and by Yuan and Wong (1995) and Schrogel and Allmansberger (1997) who apply *bgaB* as a reporter gene. In the study by Chen et al. (2008) the optimal conditions for the enzyme were found to be pH 7.0 and 70 °C. Despite the coherence between temperature optima, the pH optimum in the present study (pH 6.4) slightly deviates from that data. Unlike the study by Chen et al. (2008), all activity measurements in our study were performed directly on the cell lysate. Dong et al. (2011) utilized His-tagging of the protein for purification and found pH optimum to be 7.0. It is possible that the addition of the affinity tag may affect protein function and pH optimum, as it has previously been observed for other proteins (Thielges et al. 2011). Additionally, both studies determined pH optimum at 55 °C, while optimal temperature for this enzyme’s activity is 70 °C, the significant influence by the temperature on the activity of BgaB was also shown (at lower temperatures) by Welsch et al. (2012). The study by Schrogel and Allmansberger (1997) test cell extract and found pH optimum coherent to this study, despite that the temperature of the assay was 55 °C. Half-life of the BgaB protein was not assessed in this study, but it has been reported to be 120 h at 60 °C and 9 h at 70 °C (Chen et al. 2008). This and the temperature profile support the application of BgaB as a marker/reporter for organisms growing at higher temperatures (<75 °C).

Although *E. coli* grows optimally at a temperature where BgaB has only marginal activity, S-gal assay on solid medium is sensitive enough to produce distinct black colonies. Expression of *bgaB* in *Geobacillus* growing at 60 °C both aerobically and anaerobically similarly resulted in development of black colonies in contrast to *lacZ* from *E. coli*. This is in coherence with the study by Welsch et al. (2012). The observation points to a wide applicability of *bgaB* as a genetic marker, since it functions both in mesophiles and thermophiles, Gram-positive and Gram-negative bacteria. As shown in this study, the color development is not dependent on oxygen, unlike the most commonly used reporter systems (Piatkevich and Verkhusha 2011), such as GFP (Chalfie et al. 1994). Suzuki et al. (2013) showed its applicability in thermophilic bacteria under aerobic conditions, and here we have expanded this to include anaerobic conditions. By combining *bgaB* and S-gal we achieved a much higher sensitivity than when using X-gal as a substrate as done by Suzuki et al. (2013). This way, the *bgaB*/S-gal combination has a clear advantage for anaerobic and/or thermophilic bacteria where the availability of genetic tools is still scarce.

Generation of promoter libraries with varying strength by altering the flanking regions surrounding consensus motifs within the promoter is a well-recognized method (Gilman and Love 2016; Hammer et al. 2006; Jensen and Hammer 1998a), which was also applied for *G. thermoglucosidans* (Pogrebnyakov et al. 2017). In this study, a change in the promoter strength of fivefold was achieved, proving the applicability of the method. Using a similar reporter system restricted to mesophilic condition Jensen and Hammer (1998b) obtained a 400-fold change in activity by randomizing of the separating spacer sequences, while the consensus sequences were left intact. Selection of more clones and/or varying the consensus sequences is thus likely to result in greater viability with the respect to the activity. In general, there were no obvious features in promoter sequences, that distinguished strong and weak ones. However, slight variation in GC content between six promoters with highest (GC 31%) and lowest (26%) activity was observed. Future experiments will show the activity of the remaining promoters when expressed in *Geobacillus*. The promoter activity is likely to be different in *Geobacillus*, as promoter activity can be strain-dependent (Jensen and Hammer 1998b).

The experimental setup described above allowed us to construct a promoter library, select relevant clones, assess expression levels, and sequence the promoters within 4 days. It is expected that variation in other regions, such as Shine–Dalgarno affecting expression levels, could be assessed similarly. Since this robust method is very sensitive for identifying positive mutants, it is also highly suitable for an automated cloning and selection platform. Particularly, distinct black colonies with sharp edges are easily recognized by computer software, which facilitates improved automated colony picking. Additionally, simple equipment can be used to the perform analysis using this reporter, in contrast to the expression profiles of various libraries based on fluorescent proteins, which require analysis using fluorescent plate readers or flow cytometry.

In summary, we assessed the reporter system, focusing on application for thermophilic and anaerobic microorganisms, consisting of a thermostable β-galactosidase and its chromogenic substrate S-gal. Optimal conditions for the enzyme activity were 70 °C and pH 6.4. However, the reporter system proved sensitive over a range of different temperatures and pH values, in Gram-negative (*E. coli*) and Gram-positive (*G. thermoglucosidans*) bacteria, and under aerobic and anaerobic conditions. Thus, the reporter system presented in this study is a promising tool for fast automated high-throughput applications.

## Additional file

**Additional file 1.** Additional figures.

## Abbreviations

S-gal: 3,4-cyclohexenoesculetin β-d-galactopyranoside
X-gal: 5-bromo-4-chloro-3-indolyl-β-d-galactopyranoside
U: unit
IPTG: isopropyl β-d-1-thiogalactopyranoside
RPM: revolutions per minute
GFP: green fluorescent protein
C: celsius
USER: uracil-specific excision reagent
Abs: absorbance
V: volume
t: time
OD600: optical density, of a sample measured at a wavelength of 600 nm
LB: lysogenic broth
MU: Miller units
