# Supplementary material for: Application of the thermostable β-galactosidase, BgaB, from Geobacillus stearothermophilus as a versatile reporter under anaerobic and aerobic conditions
Source: AMB Express. 2017 Sep 6;7:169. doi: 10.1186/s13568-017-0469-z (PMC5585113; doi:10.1186/s13568-017-0469-z)

Supplementary materials

Applied Microbiology and Biotechnology

Application of the thermostable β-galactosidase, BgaB, from *Geobacillus stearothermophilus* as a versatile reporter under anaerobic and aerobic conditions

Torbjørn Ølshøj Jensen*, Ivan Pogrebnyakov*, Kristoffer Bach Falkenberg, Stephanie Redl, Alex Toftgaard Nielsen[┼].
*shared 1st authorship

The Novo Nordisk Foundation Center for Biosustainability, Technical University of Denmark, Lyngby, Denmark

[┼]Corresponding author: Alex Toftgaard Nielsen, Kemitorvet Building 220, 2800 Kongens Lyngby, Denmark, +45-45258010, atn@biosustain.dtu.dk

Fig. S1 Alignment of the promoter sequences arranged according to their activity, with the most active promoter in the top, the black boxes indicate the -35 and -10 regions of the promoter

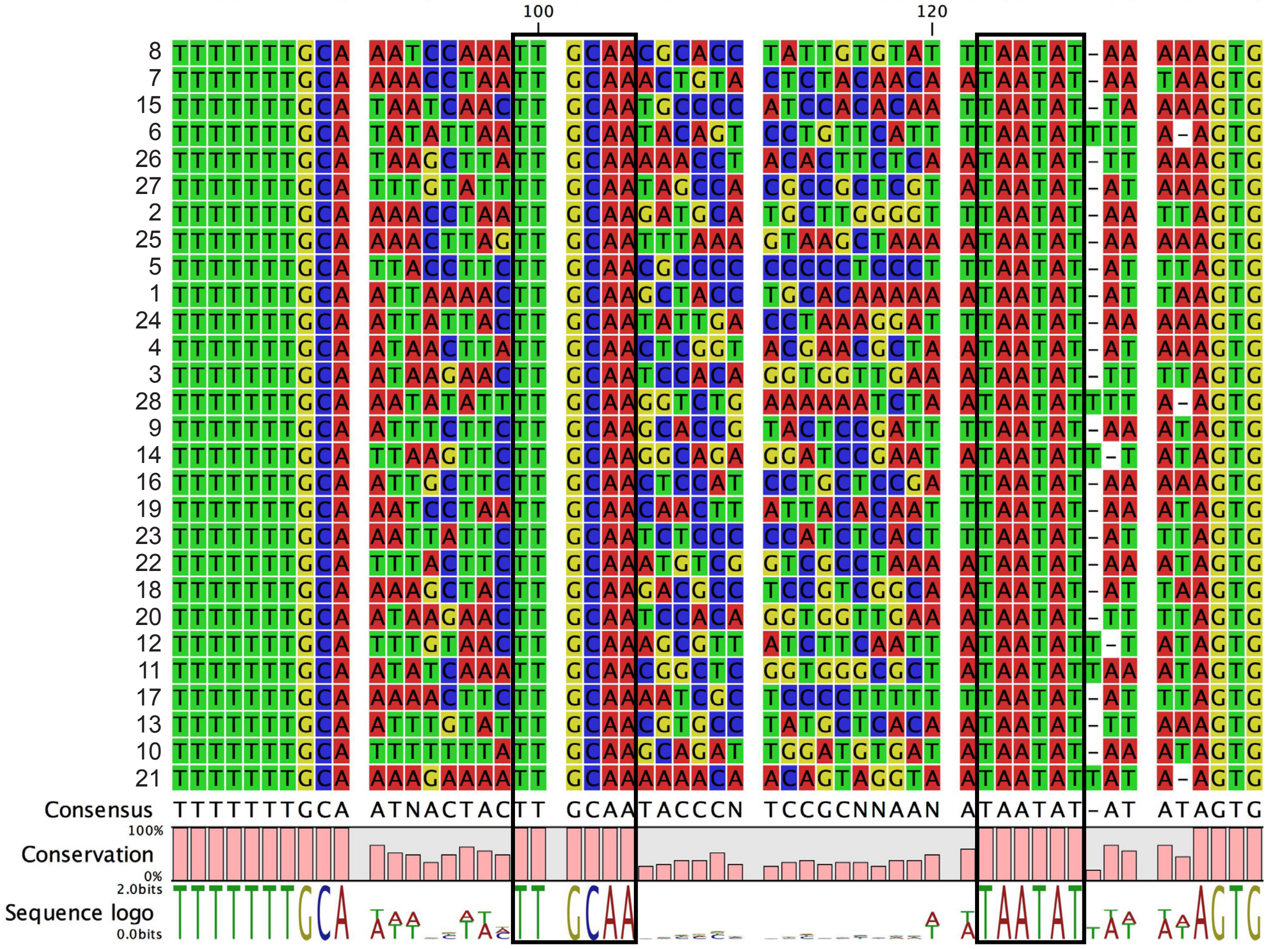

Fig. S2 Cells expressing β-galactosidase on a medium supplemented with S-gal. a, *E. coli* **TOP10 parent strain with empty plasmid and b,** *E. coli* **TOP10 with** *bgaB* **expressed from a plasmid**

a b

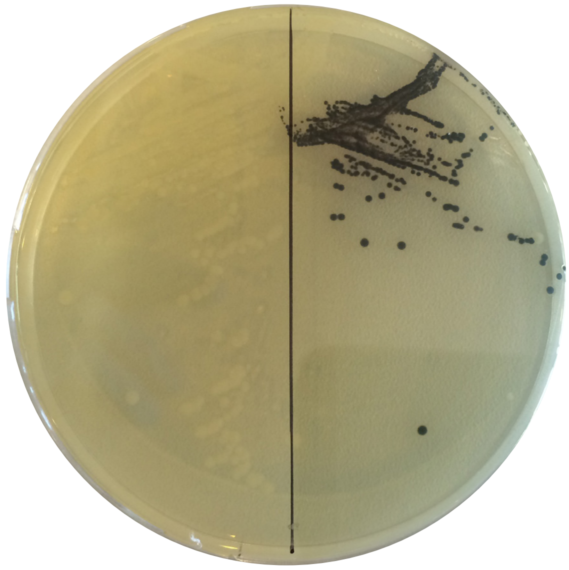

Supplement: Supplementary file 1 — Additional file 1. Additional figures. [file 13568_2017_469_MOESM1_ESM.pdf]
